# Shared burden of ultra-rare genetic variants across a spectrum of motor neuron diseases

**DOI:** 10.1186/s40035-025-00516-2

**Published:** 2025-10-29

**Authors:** Gang Wu, Wenan Chen, Joanne Wuu, Angita Jain, Jason Myers, Isabell Cordts, Evadnie Rampersaud, Jeannine M. Heckmann, Melissa Nel, Volkan Granit, Jeffrey Statland, Andrea Swenson, John Ravits, Corey T. McMillan, Lauren Elman, James Caress, Ted M. Burns, Erik P. Pioro, Jaya Trivedi, Jonathan Katz, Carlayne Jackson, Samuel Maiser, David Walk, Yuen So, Jacob L. McCauley, Matthew C. Baker, J. Paul Taylor, Stephan Zuchner, Rosa Rademakers, Marka van Blitterswijk, Michael Benatar

**Affiliations:** 1 Center for Applied Bioinformatics, St. Jude Children’s Research Hospital, Memphis, TN USA; 2 Mayo Clinic, Rochester, MN USA; 3 Department of Neurology and ALS Center, University of Miami Miller School of Medicine, Miami, FL USA; 4 Department of Neuroscience, Mayo Clinic, Jacksonville, FL USA; 5 Department of Neurology, School of Medicine and Health, University Hospital Rechts der Isar, Technical University Munich, Munich, Germany; 6 Division of Neurology, Department of Medicine and Neuroscience Institute, University of Cape Town, Cape Town, South Africa; 7 Neurogenomics Lab, Department of Medicine, and Neuroscience Institute, University of Cape Town, Cape Town, South Africa; 8 Department of Neurology, Medical Center, University of Kansas, Kansas City, KS USA; 9 Department of Neurology, University of Iowa Carver College of Medicine, Iowa City, IA USA; 10 Department of Neurosciences, School of Medicine, University of California, San Diego, USA; 11 Department of Neurology, University of Pennsylvania Perelman School of Medicine, Philadelphia, PA USA; 12 Department of Neurology, Wake Forest School of Medicine, Winston-Salem, NC USA; 13 Department of Neurology, University of Virginia School of Medicine, Charlottesville, VA USA; 14 Djavad Mowafaghian Centre for Brain Health, University of British Columbia, Vancouver, Canada; 15 Department of Neurology, UT Southwestern Medical Center, Dallas, TX USA; 16 Department of Neurology, California Pacific Medical Center, San Francisco, CA USA; 17 Department of Neurology, University of Texas Health Science Center at San Antonio, San Antonio, TX USA; 18 Department of Neurology, Department of Neurology, Hennepin Healthcare, University of Minnesota, Minneapolis, MN USA; 19 Department of Neurology, University of Minnesota, Minneapolis, MN USA; 20 Department of Neurology and Neurological Sciences, Stanford University School of Medicine, Palo Alto, CA USA; 21 John P. Hussman Institute for Human Genomics, University of Miami Miller School of Medicine, Miami, FL USA; 22 Department of Cell and Molecular Biology, St. Jude Children’s Research Hospital, Memphis, TN USA; 23 Department of Human Genetics, University of Miami Miller School of Medicine, Miami, FL USA; 24 Department of Neuroscience, Mayo Clinic, 4500 San Pablo Road, Jacksonville, FL USA; 25 Department of Biomedical Sciences, University of Antwerp, Antwerp, Belgium; 26 VIB Center for Molecular Neurology, VIB, Antwerp, Belgium

## Main text

Emerging evidence suggests an intricate genetic architecture in motor neuron diseases (MNDs), involving not only monogenic causes but also, to varying extents, risk alleles and oligogenic or polygenic contributions [1–3]. The potential for shared genetic risk across related diseases has motivated us to examine the contributions of rare variants in canonical and non-canonical disease-associated genes in a group of MNDs to understand the gap in heritability. To this end, we have leveraged a well-characterized cohort from the CReATe (Clinical Research in amyotrophic lateral sclerosis and Related Disorders for Therapeutic Development) Consortium's Phenotype-Genotype-Biomarker (PGB1) study.

**Fig. 1:**
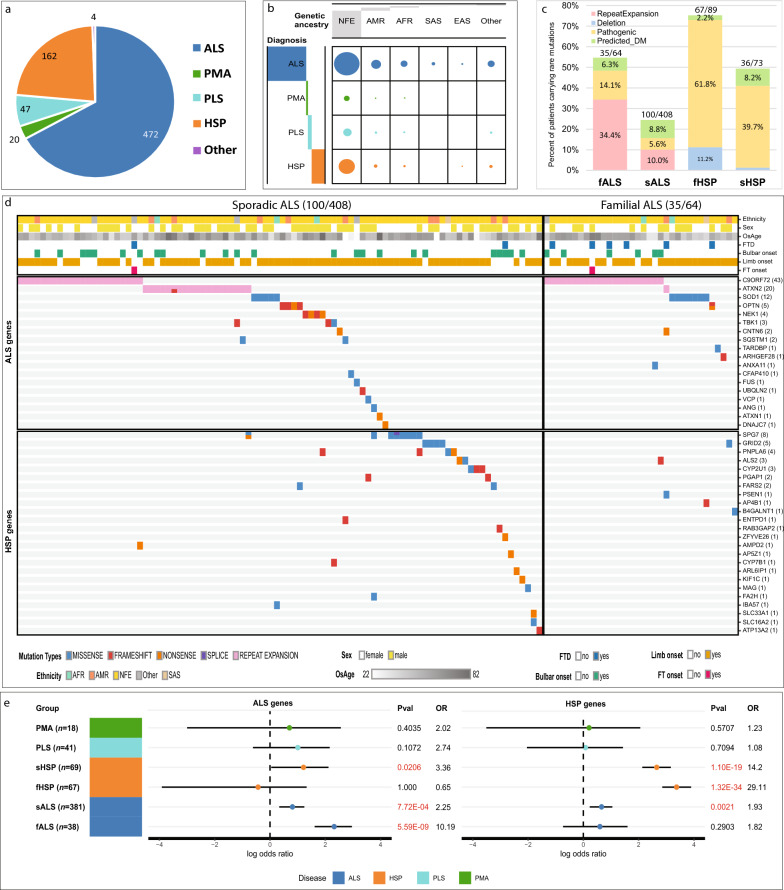
Genetic risk of rare variants in the PGB1 cohort. **a** The composition of the cohort. **b** The distribution of genetic ancestries of participants by clinical diagnosis. The grey bar reflects the relative proportion of the number of patients. **c** Contributions of URVs to familial and sporadic ALS and HSP. **d** The distribution of pathogenic URVs and predicted_DM URVs in genes associated with ALS and HSP for familial and sporadic ALS patients. **e** The log odds ratio computed based on the counts of URVs in canonical ALS or HSP genes in each group, against gnomAD non-neurological controls based on CoCoRV framework. Significant associations are in red text. EAS, East Asian; SAS, South Asian; AFR, African; NFE, Non-Finish European; AMR, American Indian; fALS, familial ALS; sALS, sporadic ALS; fHSP, familial HSP; sHSP, sporadic HSP

There are four major disease groups in this study, with ALS and hereditary spastic paraplegia (HSP) comprising the majority (90.4%) (Fig. 1a, Fig. S1). We performed whole genome sequencing to characterize the small variants and copy number variations (CNVs) (Fig. S2). The PGB1 cohort is genetically diverse—comprising 9.1% indigenous American, 4.7% African, 1.4% Asian, and 5.1% with mixed genetic background (Fig. S3, Fig. 1b, Table S1a).

For our primary analysis, we focused on patients with classic ALS (*n* = 472) and HSP (*n* = 162). For both groups, we defined individuals as “familial” by combining patients with positive family history (11.9% ALS, 53.0% HSP) and additional people identified by our relatedness analysis (1.6% ALS, 1.8% HSP). In total, the familial group included 64 individuals with ALS and 89 individuals with HSP. The remaining patients were considered sporadic.

We compiled a list of known ALS- and HSP-associated genes (Table S1b) to enable the characterization of deleterious ultra-rare variants (URV, MAF < 0.001). Our ensemble approach of multiple CNV callers identified 11 ultra-rare copy number variations affecting the coding exons of *SPAST*, *FARS2*, and *SPG7* in HSP patients (Fig. S4, Table S2a). Experimental assays were performed to measure the repeat expansion in *C9orf72* and *ATXN2* (Fig. 1c). Among familial ALS (fALS) cases, 32.8% harbored a pathological expansion in the *C9orf72* gene. Additionally, an intermediate length repeat (29 ~ 33) in *ATXN2* was present in approximately 1.6% of this group. In the sporadic ALS (sALS) group, a pathological *C9orf72* repeat expansion was identified in 5.4%, and intermediate expansions in *ATXN2* in 4.7% (Fig. 1c).

Overall, we identified 423 unique deleterious URVs (including single-nucleotide variants and indels) in disease-associated genes in 222 ALS and 134 HSP participants. Among them, 100 unique variants in 124 people were classified as pathogenic URVs based on ClinVar’s definition following panel review according to ACMG criteria with matched genes and disease etiology (Fig. S5 and S6; Table S2b). Pathogenic URVs were identified in 14.1% and 5.6% of fALS and sALS patients, and in 61.8% and 39.7% of familial and sporadic HSP cases, respectively (Fig. 1c). Except slightly lower burden of pathogenic and “computationally predicted disease-mediating URVs” (predicted_DM URVs) in fALS of American Indian, we did not observe differences across ancestries when the number of cases is sufficient to assess (Fig. S7). We found that the sALS participants with pathogenic URVs (50.35 ± 3.22 years) were 6 years younger than those without (56.6 ± 0.95 years, *P* = 0.036, Fig. S8).

Leveraging public controls in gnomAD for comparison and our recently developed tool CoCoRV [4], the overall burden of URVs in the fALS group was significantly enriched in the *SOD1*, *TARDBP*, and *ANXA11* genes (Table S3a); for sALS, URVs were significantly enriched in *TBK1*, *OPTN*, *SOD1*, *NEK1*, and *DNAJC7* (Table S3b). Applying a similar approach to the HSP cohort, the major genetic determinant was *SPAST*, followed by *SPG7*, *ATL1*, and *SPG11* (Fig. S6)*.* Burden test against gnomAD controls revealed statistically significant enrichment of URVs in *SPAST*, *SPG7*, and *WASHC5* in both familial and sporadic groups (Table S3c, d). URVs in *ATL1* were only significantly enriched in familial HSP (fHSP) but not in sporadic HSP (sHSP)*.*

We then computed the gene-set odds ratio (OR) of all canonical ALS genes harboring URVs in familial and sporadic ALS [4, 5], comparing each subgroup against gnomAD controls (Fig. 1e, Table S4a). As expected, the OR of URVs in ALS genes among fALS patients compared to controls is much higher than among sALS compared to controls (OR = 10.2 in fALS vs control, OR = 2.2 in sALS vs control). A similar trend was observed in the Target ALS cohort (OR = 4.8 in fALS vs control, OR = 2.5 in sALS vs control, Table S4b). As a negative control, we performed a permutation test in PGB1 by sampling 1000 random gene sets not related to any MND. We did not observe inflation with the permutation-based *P*-value (*P* < 0.001, Fig. S9), suggesting that the observed increased burden in ALS genes in the two ALS cohorts was not due to random chance. Compared to controls, the OR of URVs in canonical HSP genes was much higher in fHSP than in sHSP patients (OR = 29.1 in fHSP vs control, OR = 14.2 in sHSP vs control, Fig. 1e).

When considering non-canonical genes (HSP genes in ALS patients or vice versa), another 89 unique variants were classified as predicted_DM URVs due to the lack of clear evidence of known disease-gene association, and hence not meeting ACMG criteria (Fig. S5, Table S2c). Notably, over 8% of such variants contributed to sporadic cases (Fig. 1c). Close examination of these predicted_DM URVs in HSP genes revealed that the sALS carriers did not have other disease-causing pathogenic URVs (Fig. 1d, lower left panel). Among these, *SPG7* has been reported to contribute to the risk of ALS [6]. But a systematic examination on the role of HSP genes in ALS or vice versa is lacking.

Applying the same approach above to the sALS population, we found significant enrichment of URVs in canonical HSP genes when compared with gnomAD controls (OR = 1.93, *P* = 0.002, Fig. 1e, Table S4). Importantly, *SPG7* variant p.A510V was significantly enriched in sALS patients (OR = 8.5, *P* = 0.00013). Other significant HSP genes harboring multiple contributing URVs included *GRID2* (OR = 3.7, *P* = 0.026), *PNPLA6* (OR = 5.0, *P* = 0.010), *CYP2U1* (OR = 8.2, *P* = 0.007), and *PGAP1* (OR = 8.8, *P* = 0.025, Table S3b), which could contribute to ALS pathogenesis through vesicle trafficking, lipid metabolism, mitochondrial function and oxidative stress, all mechanisms that have also been implicated in the pathophysiology of ALS [7].

Given this observation, we investigated other ALS cohorts with burden tests (Table S3). In AnswerALS, when compared to AllOfUs (Table S3e), HSP gene *SPART* was enriched with rare variants (OR = 3.66, *P* = 0.014). When combining URVs in all HSP-related genes in AnswerALS, there is also a slight enrichment when compared with AllOfUs (statistically non-significant). A meta-analysis combining PGB1 ALS and AnswerALS cohorts suggested that HSP genes *ARL6IP1* (OR = 11.1, *P* = 0.015) and *PNPLA6* (OR = 2.66, *P* = 0.028) were enriched with rare variants (Table S3f). Moreover, we found that *AP4S1* was enriched in ALS patients with Non-Finish European ancestry (*P* = 0.0032, OR = 16.6, Table S3g). Interestingly, *AP4S1* was marginally significant in the burden test of ProjectMinE and significant in the meta-analysis (*P* = 0.017, Table S3h). Notably, other AP-4 family genes, typically implicated in HSP, were significantly enriched with rare variants in ALS patients as well, such as *AP4B1* (OR = 20.7, *P* = 0.0496, Table S3a). In addition, we noticed that another neuropathy AP-1 protein family, *AP1S1*, was enriched in AnswerALS when compared with gnomAD control (OR = 19.7, *P* = 0.0008, Table S3e).

Similar analyses were performed to evaluate genetic risk and the contribution of URVs in canonical ALS-associated genes in HSP. Again, the burden analysis showed a significant enrichment of URVs in ALS genes in the sHSP cohort (OR = 3.4, *P* = 0.021, Fig. 1e) but not in fHSP.

Our study of ancestrally diverse populations has revealed a few new candidate genes in which URVs contribute to disease risk in both sALS and sHSP. These results improve our understanding of the genetic overlap that exists between these closely related neurodegenerative diseases. Our novel approach using the CoCoRV rare variant analysis workflow highlights a new path to unravel the genetic architecture of these MNDs, contributing to more precise and personalized understanding of heritability for these debilitating diseases.

## Supplementary Information

Additional file 1. Supplementary methods. Supplementary results. Supplementary discussion. **Fig. S1** Distributions of genetic ancestries, age of onset, and sites of onsets by sex. **Fig. S2** The workflow of variant analyses in CReATe PGB1 whole genome sequencing pipeline. **Fig. S3** Genetic architecture of the PGB1 Cohort. **Fig. S4** Uniform coverage ensures reliable CNV detection in PGB1 WGS. **Fig. S5** The workflow of variant prioritization to identify pathogenic and predicted disease-mediating ultra-rare variants. **Fig. S6** Pathogenic and predicted disease-mediating URVs in disease associated genes in HSP patients. **Fig. S7** Pathogenic and predicted disease-mediating URVs in disease-associated genes in ALS and HSP, by ancestry background. **Fig. S8** Association of URVs of various categories to age of onset of ALS and HSP. **Fig. S9** Rare variant burden test to estimate the genetic risk conferred by URVs in randomized gene sets.

Additional file 2. **Table S1**. Study cohort, disease-related genes, sequencing and variant metrics.

Additional file 3. **Table S2**. Ultra-rare germline variants in disease-associated genes in PGB1 patients

Additional file 4. **Table S3**. Burden test of ultra-rare variants in known disease genes.

Additional file 5. **Table S4**. Burden analysis to estimate the contribution of known disease genes to a spectrum of MNDs and ALS.

## Data Availability

The genotype calls and clinical information were deposited to dbGaP (phs002844). The raw whole-genome sequencing raw data are available at NIH Cloud AnVIL, as well as part of ALS Compute (phs003184). The raw genomics data are also available at St. Jude Cloud (SJC-DS-1024). Refer to each data repository for data access.

## Abbreviations

PGB1: Phenotype-Genotype-Biomarker
MND: Motor neuron diseases
ALS: Amyotrophic lateral sclerosis
HSP: Hereditary spastic paraplegia
URVs: Ultra-rare variants
