# Supplementary material for: Shared burden of ultra-rare genetic variants across a spectrum of motor neuron diseases
Source: Transl Neurodegener. 2025 Oct 29;14:54. doi: 10.1186/s40035-025-00516-2 (PMC12570777; doi:10.1186/s40035-025-00516-2)
Supplement: Supplementary file 1 — Additional file 1. Supplementary methods. Supplementary results. Supplementary discussion. Fig. S1 Distributions of genetic ancestries, age of onset, and sites of onsets by sex. Fig. S2 The workflow of variant analyses in CReATe PGB1 whole genome sequencing pipeline. Fig. S3 Genetic architecture of the PGB1 Cohort. Fig. S4 Uniform coverage ensures reliable CNV detection in PGB1 WGS. Fig. S5 The workflow of variant prioritization to identify pathogenic and predicted disease-mediating ultra-rare variants. Fig. S6 Pathogenic and predicted disease-mediating URVs in disease associated genes in HSP patients. Fig. S7 Pathogenic and predicted disease-mediating URVs in disease-associated genes in ALS and HSP, by ancestry background. Fig. S8 Association of URVs of various categories to age of onset of ALS and HSP. Fig. S9 Rare variant burden test to estimate the genetic risk conferred by URVs in randomized gene sets. [file 40035_2025_516_MOESM1_ESM.docx]

Supplementary Information

## **Supplemental methods**

**Study cohort and sample collection**

This longitudinal study was conducted as part of the international CReATe Consortium's multi-center PGB1 study, which is registered on clinicaltrials.gov (NCT02327845). The PGB1 cohort, enrolled between 2015 and 2019, comprised a total of 715 patients from 15 clinical sites, spanning the United States, Germany, and South Africa. From this cohort, n=705 with DNA samples available for whole-genome sequencing were included in the present study. Patients were diagnosed by neurologists based on available standard diagnostic criteria. Since we included a spectrum of motor neuron diseases in our study, we relied on the clinical expertise of experienced site investigators for accurate diagnosis. Importantly, clinical diagnoses were not revised based on the results of genetic analyses. The characteristics of this cohort is summarized in Table S1A and illustrated in Figure S1.

A pedigree was constructed for each participant *(i.e*. proband), capturing any known family history of ALS, HSP, a related MND, or FTD. Familial disease was defined based on at least two biological relatives affected with ALS and/or a related disorder. Demographics, including self-reported race and detailed phenotypic data, were collected for each participant. Blood was obtained for all participants, and DNA extracted following standard procedures.

For replication purposes, we utilized independent cohorts of N=223 ALS (including ALS-FTD) patients from Target ALS (<https://www.targetals.org/>)(1), and N=626 ALS patients from AnswerALS ((2)). In addition, we included the summary statistics from ALSdb ([alsdb.org](https://alsdb.org/)) and Project MinE (http://databrowser.projectmine.com/) in the meta-analyses ((3, 4)). For rare variant burden analysis using CoCoRV framework, besides the gnomAD database (https://gnomad.broadinstitute.org/) as a public control (5), we created the summary statistics for the stratified main subpopulations from the AllOfUs database (<https://researchallofus.org/>) (6).

**Disease gene curation**

An extensive list of 55 ALS- and 98 HSP-associated genes was developed based on the literature and standard databases, including the Online Catalog of Human Genes and Genetic Disorders (OMIM), Human Gene Mutation Database (HGMD), ALS online Database (ALSoD, https://alsod.ac.uk/), and Kyoto Encyclopedia of Genes and Genomes (KEGG, https://www.genome.jp/kegg/disease) (Table S1B).

## We also compiled an expanded list of motor neuron related genes by combining all genes from various resources including malacards (https://www.malacards.org/), KEGG, MDS (https://www.movementdisorders.org/MDS/About/Committees--Other-Groups/MDS-Task-Forces/Task-Force-on-Nomenclature-in-Movement-Disorders.htm), HGMD, ALSoD, GHR, OMIM, and LMD for ALS, HSP, FTD, PMA, PLS, SMA, dHMM, LS, and MSP (Table S1C). This expanded list was only used when randomly selecting non-MND genes in the permutation calculation for CoCoRV to test if enrichment of URVs in HSP genes in sporadic ALS is due to random chance.

**Analysis of repeat expansions**

Using genomic DNA, all samples from study participants were analyzed for *C9orf72* and *ATXN2* expansions. The *C9orf72* gene was screened using FAM-labeled genotyping and repeat-primer assays as previously described by DeJesus-Hernandez *et al*.(7) *ATXN2* was screened by PCR amplification over the expanded region with primers. The fluorescently labeled amplicons were run on an ABI3730XL and visualized using Genemapper software (Thermofisher, Foster City, CA).

**Whole genome sequencing and variant calling**

Whole genome sequencing (WGS) was performed at the HudsonAlpha Institute for Biotechnology Genomic Services Laboratory (Huntsville, AL), using Illumina HiSeq X10 (N=455) and NovaSeq (N=250) sequencers to generate an average of 938 million paired-end reads, each of 150 base pairs in length. The raw sequencing reads were mapped to reference human genome GRCh38. The mapping summary statistics such as genome-wide and exonic average coverage were generated with Qualimap (8). The QC metrics of WGS were summarized in Table S1D.

Variants were called following the Genome Analysis Toolkit (GATK) best practices workflow.(9) Minimum genotype quality (≥20), minimum coverage depth (≥7), and minimum variant allele frequency (≥20%) thresholds were applied using bcftools.(10) (Figure S2). The quality of variants called was summarized in Table S1E.

**Variant, sample and cohort level QC**

Quality control (QC) was performed to assess level of missingness across variants and samples, deviation from Hardy Weinberg Expectation (HWE), sex discordance between reported and genetically determined sex, and ancestry comparisons using ethnic groupings from the 1000 Genomes cohort (11). Variants were excluded if the missing call rate exceeded 0.05 and/or HWE p-value < 1x10-7. Linkage disequilibrium (LD) filtering was performed using PLINK’s indep-pairwise command, with a window size of 50 variants, a step size of 5 variants, and an r2 threshold of 0.3. Samples where genetic sex and reported sex differ were evaluated by clinicians and only genetic sex was used in the downstream analysis.

**Inference of genetic ancestry**

To infer the ancestry from the actual genotype calls, we applied the random forest classifier trained based on 1000Genome reference population (n=6, NFE, FIN, AMR, AFR, EAS, SAS) (Figure S2). The variants were first filtered using genotype quality (≥ 20), read depth (≥ 10), genotype missingness (< 0.01), and minor allele frequency (≥ 0.001). Data were then merged with the 1000 Genomes GRCh38 samples (N=2,504) to estimate genetic ancestry. Variants with MAF<0.1 in the combined 705 cohort were removed. Then the genotypes were pruned using plink with the R^2^ threshold 0.1, resulting 969,320 variants. We applied the minimum score threshold of 0.6 for the max score of each ancestry to define the main ancestry for each patient.

**Relatedness analysis**

Initial relationship inference was run using the KING-robust algorithm (12), and used to identify a set of mutually unrelated individuals in the final estimation. Using the LD-pruned data, the R package GENESIS (13) was implemented to perform population structure inference that is robust to known or cryptic relatedness (PC-AiR) and to perform relatedness estimation in the presence of population structure and admixture (PC-Relate) (12). Using only the variants in approximate linkage equilibrium, genetic sex was estimated using the X chromosome inbreeding coefficients (F), where F > 0.8 yield male calls and F < 0.6 yield female calls.

**Admixture analysis**

The program Admixture was then run on the cleaned plink file (14). Five clusters were used, and 20 runs were performed. Then CLUMPAK (15)was used to merge the 20 runs. The averaged proportions from 20 runs were used as the final proportion for each ancestry cluster (Figure S3B).

**Germline copy number analysis**

Given the complexity of human genome and challenges to accurately call germline structural variants, an ensemble approach has been employed to characterize the germline copy number variations (CNVs: amplification and deletion). For germline copy number alterations, we mainly relied on CNVnator segmentation calls (16), supplemented with CONSERTING (single sample mode, (17)) detected CNVs (Figure S2). We required at least two discordantly mapped reads (at least 1kb aways for the mated pair reads) supporting a detected segment, using an in-house filtering perl script. The frequency of copy number variations was independently determined based on in-house whole-genome sequencing data from 12,070 non-MND individuals (9,856 pediatric cancer patients, 912 sickle cell disease patients and 726 healthy individuals), hosted on St. Jude Cloud. (18). The called CNVs were overlapping with the coding exons of the protein coding genes. The ultra-rare copy number changes (completely absent using in-house non-MND WGS database) affecting the coding exons of candidate genes within each disease were further inspected to rule out the possibility of uneven low coverage in problematic regions (e.g. GC-rich first exons and repetitive regions). For instance, the GC rich first exons often showed false positive copy number deletions in Novaseq due to reduction in the coverage depth. Coverage depth tracks were provided to support the rare copy number variations affecting disease associated genes ((Table S2A, Figure S4).

**Prioritization of genetic variants**

Frequencies of identified variants were evaluated based on population frequency databases (gnomAD v3, ExAC, and 1000Genome). For copy number variants, we used gnomAD SV v2.1 (liftOver to GRCh38) and an in-house genetic database (St. Jude). Variants were classified as ultra-rare when thresholds of minor allele frequencies < 0.001 were reached across all common databases (gnomAD non-neurological exome subset population, ExAC, 1000Genome). Common databases with potential clinical relevance (OMIM, HGMD, ClinGen, Clinical Variant Database, and InterVar [2022 June 13 updates]) were used for assessing the potential pathogenicity of variants. Additionally, *in silico* prediction scores (Database for Non-Synonymous Functional Predictions, Loss-Of-Function Transcript Effect Estimator, Combined Annotation Dependent Depletion, and Splicing Variant Annotation and Interpretation) were applied. Overall, each URV was assigned to one of the six flags in a sequential order (Figure S5). Of note, the first of the pathogenic/likely pathogenic variant prioritization was only computationally assigned based on the database matches or functional annotations. Variants were assigned as pathogenic URVs when the gene (e.g., ALS-related gene) and the diagnoses (e.g., ALS) matched as well as expert panel review following American College of Medical Genetics (ACMG) criteria (Table S2B). Otherwise, they were defined as “computationally predicted disease-mediating URVs” (Table S2C).

**Rare variant burden analysis**

A rare variant burden test against gnomAD summary frequency estimates was performed separately for familial and sporadic ALS and HSP patients in PGB1 cohort, respectively. For the familial cases, only the family member with the earliest age of onset per family was used to avoid overcounting. Rare variant burden analysis between cases in the PGB1 cohort and non-neurological controls from gnomAD was performed for all disease-associated genes using a previously described framework: the consistent summary counts based rare variant burden test (CoCoRV)(19) (Table S3). In this framework, instead of using the full genotype of each variant from the patient and control cohort, it applies the same variant filtering criteria to retain the rare variants within each genetic ancestry of the case and control to control the diverse genetic background. More specifically, the ancestry group of each participant was inferred using the gnomAD ancestry classifier and then matched to gnomAD controls of the same ancestry group. Coverage summary statistics were calculated and only regions with a depth ≥ 10 for at least 90% of the case cohort and the control cohort were considered. Variants having allele frequency ≤ 0.001 in both the case cohort and gnomAD (max reference population) were further analyzed. The number of individuals carrying the prioritized rare germline variants in each gene were summarized as 2 by 2 contingency tables in each genetic ancestry for case and controls (Table S3), subjected to Cochran–Mantel–Haenszel test (CMH) tests.

When assessing the association of a set of genes (e.g., ALS-associated or HSP-associated), we limited our analysis to the coding sequence regions of the specified genes. Specifically, we used gene annotation from GENCODE and kept the coding sequences of each gene plus the flanking region up to 6 nucleotides to include canonical splicing sites. The defined region was then treated as a single “gene set,” and a count-based rare variant burden test was used to assess the association. For an individual carrying more than one pathogenic or computationally predicted disease mediating URV in disease associated genes (regardless of autosomal dominant or autosomal recessive inheritance), it was counted only once in the final test. We also limited our analysis to point mutations and indels, because the sample size of the gnomAD CNV/SV database is different and there is no information on *C9orf72* and *ATXN2* repeat expansions in the public controls.

For replication of findings, rare variant burden tests were performed for TargetALS against gnomAD, and AnswerALS against AllOfUs using CoCoRV framework. For ALSdb NFE summary statistics for all genetic variants reported, a Fisher’s exact test was performed against gnomAD v2.1 NFE summary statistics (Table S3).

**Gene-set-based rare variant analysis.**

When assessing the association of a set of genes, such as known ALS or HSP genes, we used a bed file to define the coding sequence regions of the specified genes. Specifically, we used gene annotation from the GENCODE v35 (20) GRCh38 GTF file and kept the CDS regions of each gene plus the flanking region up to 6 nucleotides to include canonical splicing sites. Then the defined region was treated as a single “gene set” and CoCoRV was used to assess the association (19) (Table S4). To check whether there was inflation in the computed p-values, we randomly sampled 60 genes from chromosome 21 excluding motor neuron disease related genes (Table S1B) and assessed the significance of the random gene set. We repeated the random sampling of 60-gene set 1000 times. The p-values from randomly sampled gene sets were also used to calculate an empirical p-value for each specified gene set as

$p_{empirical}=\frac{\#(p_{random}\leq p_{specified})+1}{\#repeat+1}$.

A Q-Q plot was generated to evaluate the inflation of the burden test (Figure S9).

**Statistical analysis**

The statistical significance (p-value) of HSP and ALS genes was reported based on CoCoRV pipeline. In addition, a binomial test was used to assess potential sex bias. Where applicable, the p-values were adjusted for multiple testing using the Benjamini and Hochberg method. Meta-analysis was performed for the CoCoRV test for PGB1 ALS and Answer ALS by concatenating the ancestry stratified 2x2 tables and then performing CMH test as described in CoCoRV. For meta-analysis of rare variant burden test results of ALSdb ([alsdb.org](https://alsdb.org/)) (3)and ProjectMinE (http://databrowser.projectmine.com) ((4)), we used Fisher’s p-value combination method.

## **Supplementary results**

**Whole genome sequencing and mapping**

DNA fragments were subjected to size selection (minimum of 300bp) to ensure high quality sequencing outcome, leading to an average of empirical library insert size of 333bp (± 35.6bp) (Table S1D). We achieved an average of 40X (±8X) genome wide coverage depth (Table S1D). The average mapping rate for all 705 samples was 99.7%, with low duplication rate (<10%) (Table S1D). Most samples have over 90% of exonic regions covered more than 20X, ensuring high confidence genotyping calling for point mutations and small indels, as well as accurate characterization of structural variants and copy number changes (Table S1E).

**Cohort characterization: sex bias**

Of the 705 PGB1 participants included in this study, 472 (67%) had ALS (450 classic ALS, 22 ALS-FTD); 162 (23%) HSP; 47 (6.7%) PLS; 20 (2.8%) PMA; and 4 (0.5%) other diagnoses (2 multisystem proteinopathy, 1 adult-onset spinal muscular atrophy, 1 FTD) (Table S1A, Figure S1A). The cohort had a male preponderance (387/705, 54.9%, p=0.010), most notably among patients with ALS (271/472, 57%, p=0.002) and PMA (13/20, 65%, p=0.005,). The male preponderance in ALS is more obvious in patients of European ancestry (Figure S1B). The mean (±SD) age at onset in the ALS group was 56.5 (±12.7) years, whereas it was 32.3 (±18.1) years in the HSP group – consistent with known ranges of age of onset (Table S1A, Figure S1C). Interestingly, the male bias in ALS was again observed mainly for patients with age of onset between 40 to 60 years old (Figure S1D). By contrast, slightly higher percentage of female HSP patients were observed for the age of onset between 40 to 50 years old (Figure S1D). In terms of sites of onset, higher percentage of ALS patients were of limb onset (Figure 1F).

**Cohort characterization: genetic ancestry**

Principal component and admixture analyses, with reference populations from the 1000Genome Project, indicated admixed ancestries in our cohort, especially in the ALS group (Figure S3A, S3B). The genetically inferred ancestral background was further determined based on genotype calls from whole-genome sequencing for each patient, using a random forest classifier trained with the reference populations from the 1000Genome Project (Figure S3C). It showed that the study cohort was genetically diverse – comprising 9.1% Indigenous American, 4.7% African, 1.4% Asian, and 5.1% with mixed genetic background (Figure S3, Table S1A). Genetically inferred ancestral background was also largely consistent with self-reported race; ~10% of the 618 self-reported White patients were, however, genetically inferred as Indigenous American (n=52 [8.4%]) or “Other” (n=13 [2.1%]) (Figure S3C).

**Cohort characterization: self-report and genetics confirmation of family history**

One hundred and forty-seven (21%) patients (56 ALS, 86 HSP, 3 PLS, 1 FTD and 1 PMA) self-reported a familial history of ALS or a related disease. Relatedness analysis using genotype calls identified another 12 patients (8 ALS, 3 HSP and 1 PLS) as being related (up to 2^nd^ degree of relatedness). For analytic purposes, we regarded these 159 (22.6%) self-reported and intra-cohort related participants as ‘familial’, and the remaining 546 as ‘sporadic’. While over half of our HSP participants (89/162, 54.9%) are familial, only ~14% (64/472) of ALS participants self-reported or genetically determined positive of family history. There is a slight female preponderance (52/89, 58.4%) in the familial HSP (fHSP) group, but no sex bias was observed for familial ALS (32/64, 50%, fALS).

**Genomic characterization of deleterious URVs in known disease-associated genes**

With high-depth whole-genome sequencing, we investigated the potential contribution of structural variation to the disease. We employed an ensemble approach for copy number variation calling, followed by post calling filtering based on supporting discordant reads (Figure S2). We only detected 11 ultra-rare copy number variations in known disease associated genes in HSP patients but not in ALS patients (Table S2A, Figure S4). The size of these deletions ranged from ~3KB to ~2MB. Notably, none were found in the gnomAD or St. Jude in-house structural variation databases. A total of 11.2% of familial HSP had identified deletions, compared to only 1.4% in the sporadic subgroup (Figure 1C, Table S2A).

Among the full ALS cohort (combining familial and sporadic cases), pathogenic URVs in canonical ALS genes and computationally predicted disease-mediating URVs in non-canonical disease-related genes were identified in 15.3% of patients (Figure 1C). Mutations in *SOD1* accounted for 10% of the familial ALS cases, whereas only 1% of sporadic ALS cases harbored pathogenic SOD1 URVs (Figure 1D, Table S2A). Other, less common, pathogenic URVs or predicted disease-mediating URVs in familial and sporadic ALS cases were also detected (Figure 1D).

Analysis of the full HSP cohort revealed pathogenic URVs in 51.9% and computationally predicted disease-mediating URVs in 4.9% (Figure 1C). Pathogenic URVs in *SPAST* were identified as the major genetic determinant of both familial HSP (50.6%) and sporadic HSP (19.2%), followed by URVs in SPG7, ATL1, and SPG11 (Figure S6).

**Burdens of rare variants in ALS and HSP patients of different genetic ancestries**

Given the diverse background of ALS patients in the PGB1 cohort, we also assessed the frequency of URVs among different ancestral groups. In the familial ALS group, we observed a slightly lower fraction of patients with indigenous American and mixed genetic backgrounds carrying pathogenic or predicted disease-mediating URVs than those of other ancestral backgrounds. In the sporadic ALS group, however, the percentage of patients carrying pathogenic or computationally predicted disease-mediating URVs was comparable (~20%) across ancestral groups (Figure S7A). Unlike ALS, the relative frequencies of pathogenic or computationally predicted disease-mediating URVs among familial and sporadic HSP patients did not differ based on ancestral background. (Figure S7B).

**Association of age of onset with status of rare variants in ALS- and HSP-related genes**

With the characterization of pathogenic URVs, we evaluate their contribution to the age of disease onset. PGB sALS patients with pathogenic URVs (average age of onset: 50.3 yrs) were at least 6 years younger than the sALS patients of other categories (P<0.03, one-tailed t-test, Figure S8). Similar trend was observed in sALS patients in TargetALS (Figure S8). We didn’t observe clear differences in age of onset between HSP patients with and without a pathogenic URV (Figure S8).

**Burdens of rare variants in sporadic PLS and PMA patients**

Similar procedure was applied for sPLS and sPMA using both ALS and HSP-related gene sets. However, there is no increased genetic burden of URVs in ALS genes or HSP genes in these two diseases compared with healthy controls in gnomAD (Figure 1E, Table S4A), which is likely due to the small sample size and/or heterogeneous underlying genetic architecture in sporadic PLS and PMA.

**Supplementary discussion**

In this study, we took advantage of the unique strengths of the CReATe PGB1 study cohort, which comprises clinically well-characterized, multi-ancestral patients from a wide spectrum of MNDs. This enabled us to delineate overall patterns of genetic architecture across the spectrum of these related disorders.

We performed a comprehensive characterization of ultra-rare germline single-nucleotide variants and small indels with multi-tiers of deleterious mutations. Our focus was on pathogenic URVs and computationally predicted disease-mediating URVs in known disease-associated genes, with reference to disease subgroups and among patients from diverse genetic ancestral backgrounds. In the ALS cohort, the frequency of causative C9orf72 and SOD1 pathogenic variants was consistent with what has been reported in the literature(21). We observed higher frequency of ATXN2 expansion in sALS than fALS, consistent with previous reports (22-24). We also observed a relatively low frequency of pathogenic variants in TARDBP, FUS, VCP, TBK1, and NEK1. Similarly, in the HSP cohort, pathogenic variants in SPAST and ATL1 were fairly frequently observed, as has been reported in larger populations. Interestingly, unlike the HSP cohort, no ultra-rare copy number variations were detected in the ALS cohort for the disease associated genes examined in this study.

Given the phenotypic overlap between these diseases, particularly regarding HSP and ALS with upper motor neuron involvement, studying shared mechanisms of motor neuron degeneration is highly relevant. Neuropathological studies have suggested that, in HSP, the primary pathological mechanism is a ‘‘dying-back’’ axonal neuropathy, primarily affecting the upper motor neurons of the corticospinal tracts.(25) Importantly, numerous HSP-linked genes are related to axonal transport and intracellular trafficking, highlighting the vulnerability of long motor neuron axons to alterations in the axonal transport machinery.(26) Of note, in ALS, there is also growing evidence that the motor neuron pathology begins at distal axons and proceeds as a “dying-back” phenomenon, and disturbances in axonal transport are key pathological events contributing to many monogenetic forms of ALS.(26, 27) Furthermore, it has previously been suggested that variants in genes primarily associated with HSP – such as SPG7,(28) SPG11,(29) KIF5A,(30, 31) ERLIN2,(32) ALS2,(31) and UBQLN2(33) – have also been observed in ALS patients, and vice versa. It was interesting to note that despite the significant phenotypic overlap between HSP and PLS, no genetic association was noted with PLS although the sample size was limited. However, our study provides, for the first time, genome-wide statistical evidence for genetic overlap between ALS and HSP. Our observed enrichments of pathogenic and computationally predicted disease-mediating URVs in disease-associated genes, particularly in sporadic individuals, indicates a significant shared genetic risk between these two diseases. Notably, we found multiple heterozygous SPG7 pathogenic variants in our cohort of ALS patients.(28, 34) We also observed that SPG7 p.A510V might increase disease risk in the sporadic ALS subgroup, though this requires validation in larger independent cohorts. Meta-analysis of the PGB1 cohort and AnswerALS suggested that canonical HSP genes ARL6IP1 and PNPLA6 could also increase the risk of ALS. Moreover, we identified multiple AP-4 complex genes, especially AP4S1, with enriched URVs in ALS, which was observed in multiple independent ALS cohorts.

It has been suggested that the occurrence of ALS is consistent with a multistep process.(35, 36) Individuals carrying a pathogenic variant may or may not develop the disease; and if they become affected, they often develop symptoms later in life even though the variant is present from birth. The number of steps necessary for developing the disease in the absence of a mutation is proposed to be six; this number however, is lower for high-penetrance mutations (e.g., in the SOD1 gene). ALS susceptibility is also influenced by other disease-associated genes acting as risk factors. The interplay of various genetic and environmental factors might lead to a cascade of events, thereby gradually increasing the risk of developing ALS, accounting for step(s) to this multi-step process. For example, studies have revealed that the length of a repeat expansion in the HTT gene, known to cause Huntington’s disease, can influence ALS risk.(37) Similarly, HSP genes in ALS might represent one or more steps in this multistep process, influencing key cellular functions that contribute to motor neuron degeneration.

On the other hand, given the complexity of HSP genetics, with suggestions of involvement extending beyond Mendelian inheritance, a similar multi-step process could potentially be applied to HSP. If so, it remains to be determined how many steps are needed for developing HSP. This multistep process also encourages the use of innovative approaches, such as those that we have employed, in the search of additional risk genes, which will contribute to our understanding of the missing heritability in these diseases.

We have characterized loss-of-function rare copy number variations for known disease-associated genes. Interestingly, in our cohort, we only observed pathogenic rare germline copy number variations in HSP patients – specifically, deletions in the SPAST gene, the most commonly associated HSP gene(38, 39) – but not in ALS patients. This finding could be attributed to the fact that, in general, copy number variations are more commonly associated with HSP, representing about 20% of the disease-causing variants as reported in multiple patient cohorts.(40-42) In contrast, our approach didn’t uncover ultra rare copy number variations for disease associated genes in ALS, which appear to be less common and possibly understudied.(43-45) In addition to the modest sample size of our ALS cohort, technological limitations could be another factor might have hindered our ability to effectively detect rare copy number variations or other types of structural variation due to. For example, the characterization of repeat expansions in C9orf72 and ATXN2 was conducted separately, given the constraints of short-read sequencing at the time the study was performed. Due to the lack of C9orf72 and ATXN2 repeat expansion status in public gnomAD control database, we were not able to integrate this into our estimation of mutational burden, which could be an interesting question for a large case/control cohort study where these types of genomic variants could be measured in all individuals.

**Supplementary Figures**


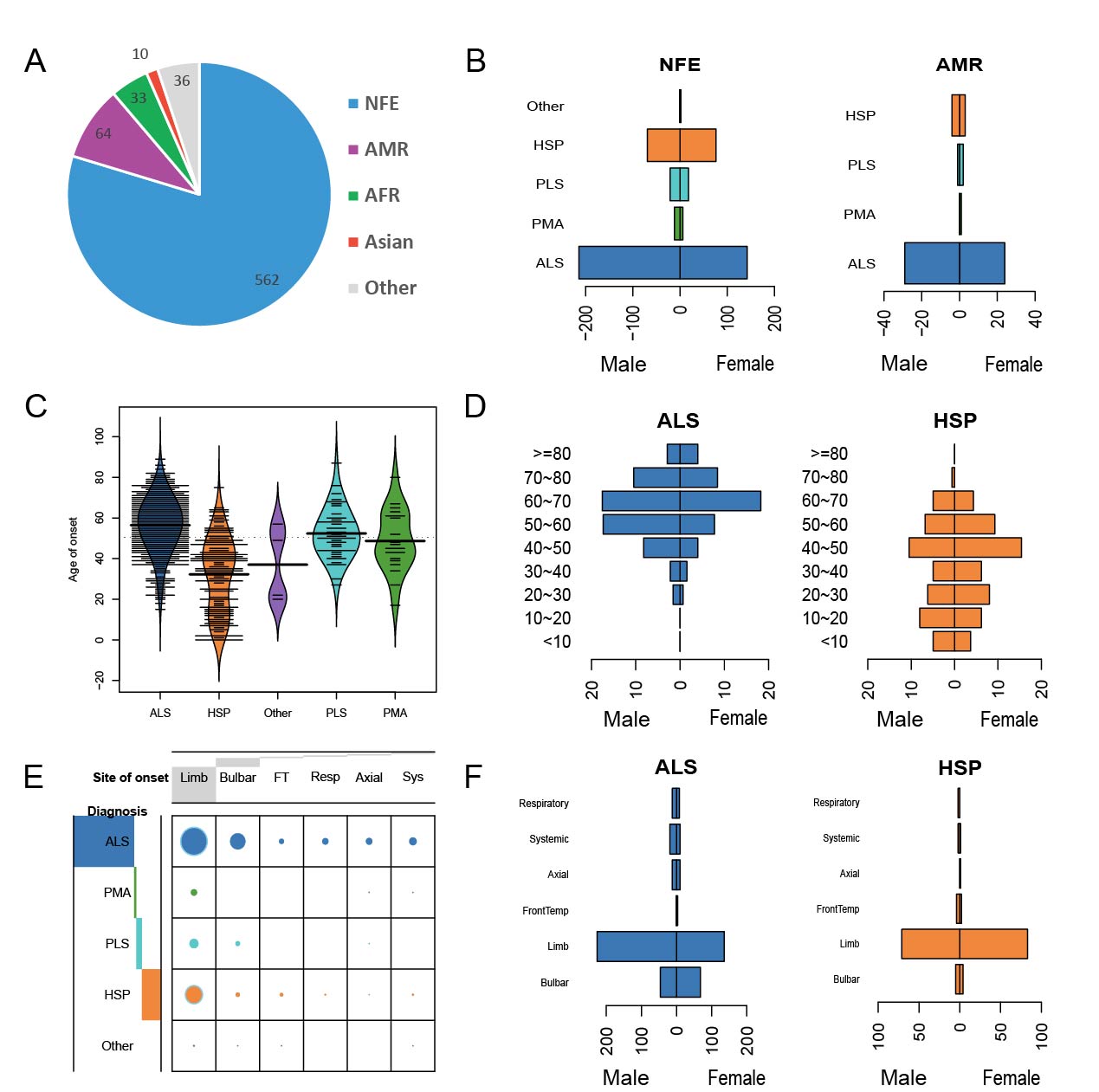


**Figure S1. Distributions of genetic ancestries, age of onset, and sites of onsets by sex.** A and B, genetic ancestries determined by admixture analysis; C and D, age of onsets; E and F, sites of onsets. AFR – African; AMR – American Indian (Latino); NFE – Non-Finish European.

**Figure S2. The workflow of variant analyses in CReATe PGB1 whole genome sequencing pipeline.** Copy number variation (CNV) analysis was performed using an ensemble approach by combining results from CNVnator and CONSERTING, both of which were based on read depth. The called CNVs were filtered with discordant read supports. Instead of annotating each CNV with in-house database, we identified CNVs that affect the coding exon of protein-coding genes, which helps identify deleterious CNVs. For candidate genes scrutinized in this study, no filtering of population frequency was applied. If coding exons of disease-associated genes were affected by the CNVs, the variants were kept. For SNV and indels, GATK4 best practice was applied to perform genotype calling and joint calling. Following the VQSR quality filtering and annotation, high-quality SNV/indels were kept for downstream analyses. The population frequency for each rare variant was annotated with gnomAD database. For disease-associated genes curated in this study, further filtering was done to prioritize ultra-rare highly deleterious variants.

**Figure S3. Genetic architecture of the PGB1 Cohort. (A)** Overlay of the overall genetic background of PGB1 participants onto the genetic reference map determined by the first two principal components based on 1000Genome populations. **(B)** Admixture analysis with 1000Genome reference populations showing the percentage of genetic markers from different ancestral groups for each PGB1 participant. **(C)** Concordance of self-reported race and genetically inferred ancestry based on 1000Genome random forest classifiers


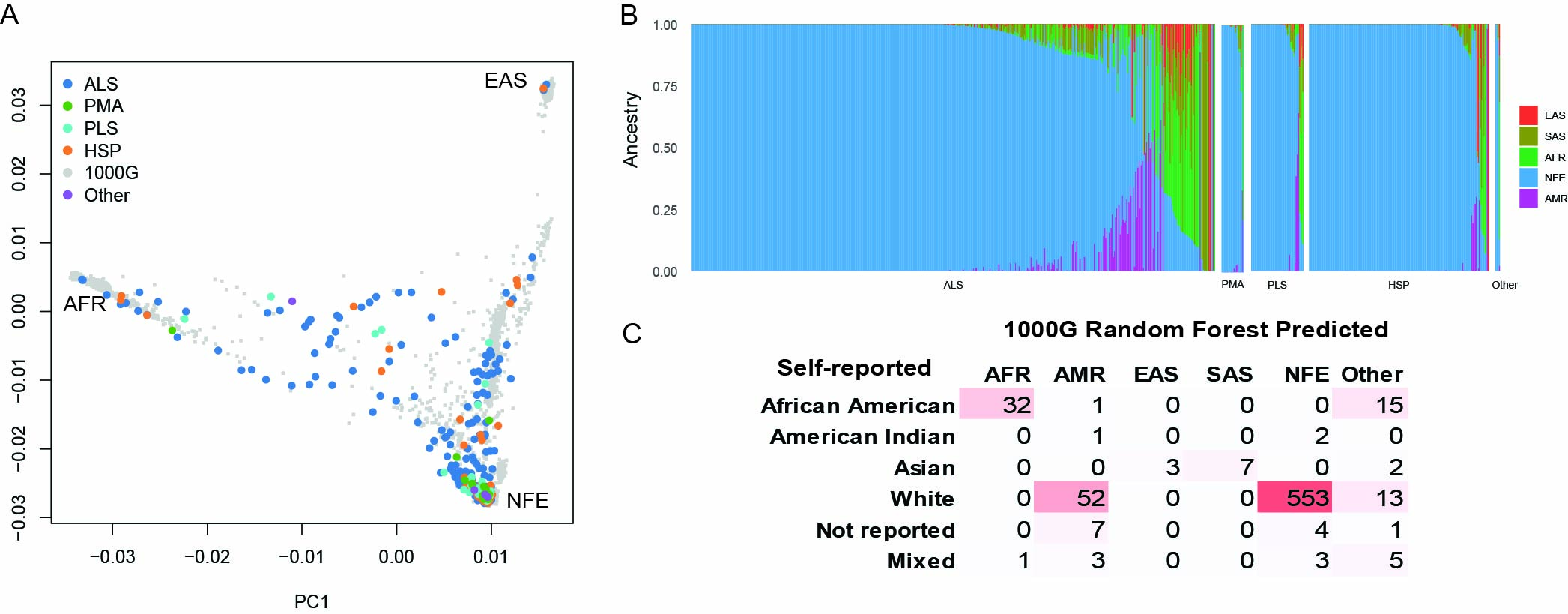


**Figure S4. Uniform coverage ensures reliable CNV detection in PGB1 WGS. (A)** Large *SPAST* heterozygous deletions in two HSP patients; **(B)** Focal *SPAST* heterozygous deletions in HSP patients; **(C)** A large *FARS2* heterozygous deletion in an HSP patient; **(D)** A focal *SPG7* heterozygous deletion in an HSP patient. All illustrated deletions in the black boxes showed a reduction of coverage depth compared with upstream and downstream diploid genomic regions.

**Figure S5. The workflow of variant prioritization to identify pathogenic and predicted disease-mediating ultra-rare variants.** Briefly, a URV will be assigned one of the six flags in the priority shown. Note that the P/LP at this step is only computationally assigned based on the database matches or functional annotations. Then a variant in the first 5 categories will be evaluated if it matches the known disease diagnosis and subject to expert panel review. Only matched diagnosis associated with the variants and meeting ACMG criteria for P/LP variants will be called “Pathogenic URV”. Otherwise, it would be remained as “computationally predicted disease-mediating URV”, as they are strong candidates for likely pathogenic rare variants. “Splicing high impact” was determined based on the spliceAI prediction. “Other high impact missense URV” refers to missense URV with REVEL scores >0.65 or CADD score >20. P: pathogenic; LP: likely pathogenic. ClinVar P/LP: defined as pathogenic or likely pathogenic in ClinVar database; ClinVar Conflicting P/LP: defined as conflicting pathogenic or likely pathogenic in ClinVar database; InterVar P/LP: computationally predicted as pathogenic or likely pathogenic by InterVar based on ACMG guidelines.

**Figure S6. Pathogenic and predicted disease-mediating URVs in disease associated genes in HSP patients.** The heatmap shows the distribution of pathogenic and computationally predicted disease-mediating URVs, in genes associated with ALS and HSP, among familial and sporadic HSP patients. HSP patients with neither pathogenic nor predicted disease-mediated URVs in these genes are not shown. Numbers in parentheses adjacent to gene names on the right-hand side of the figure indicate the number of participants in which the URV was identified. EAS – East Asian; SAS – South Asian; AFR – African; NFE – Non-Finish European; AMR – American Indian; FT onset – frontotemporal onset.


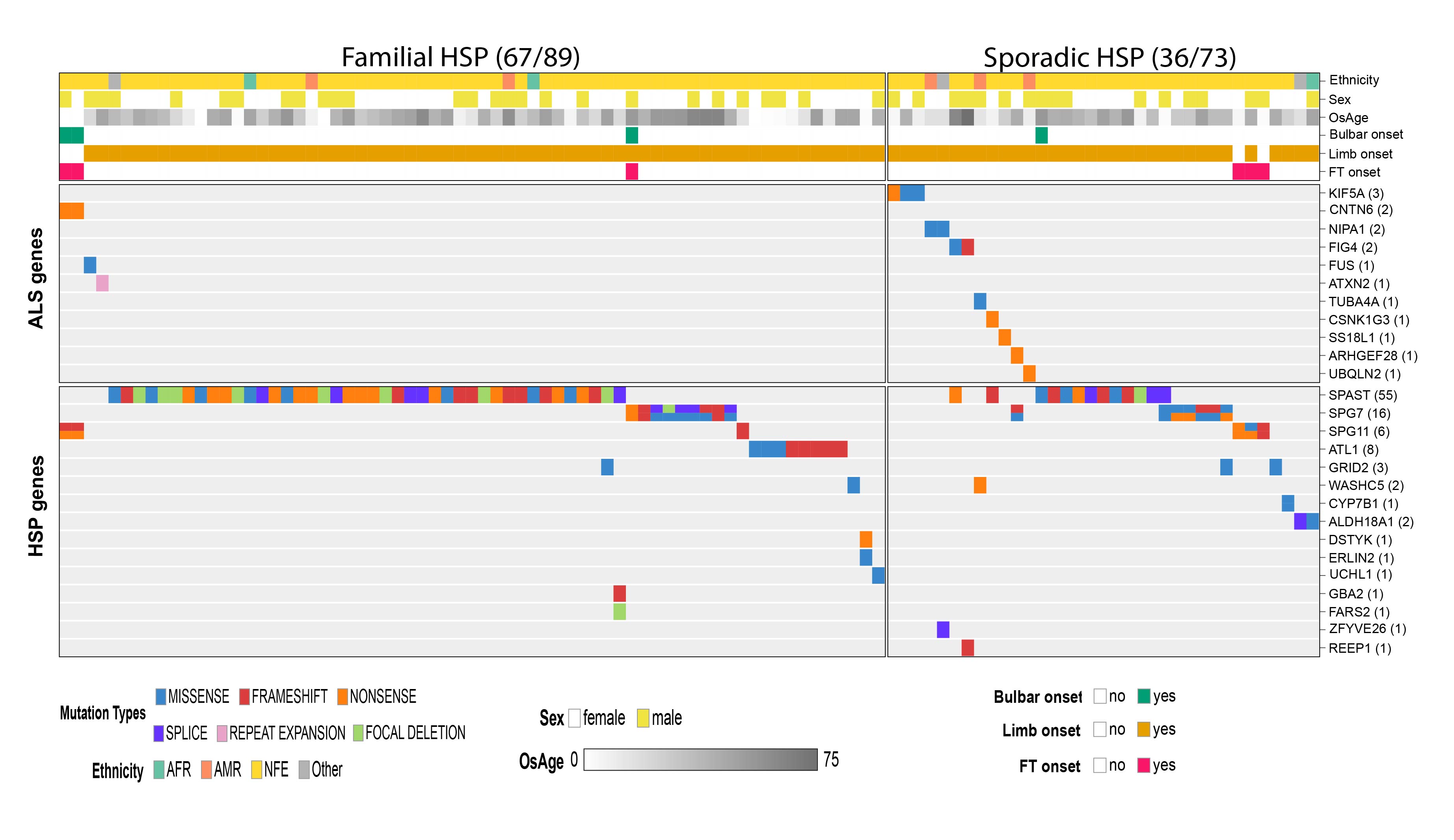


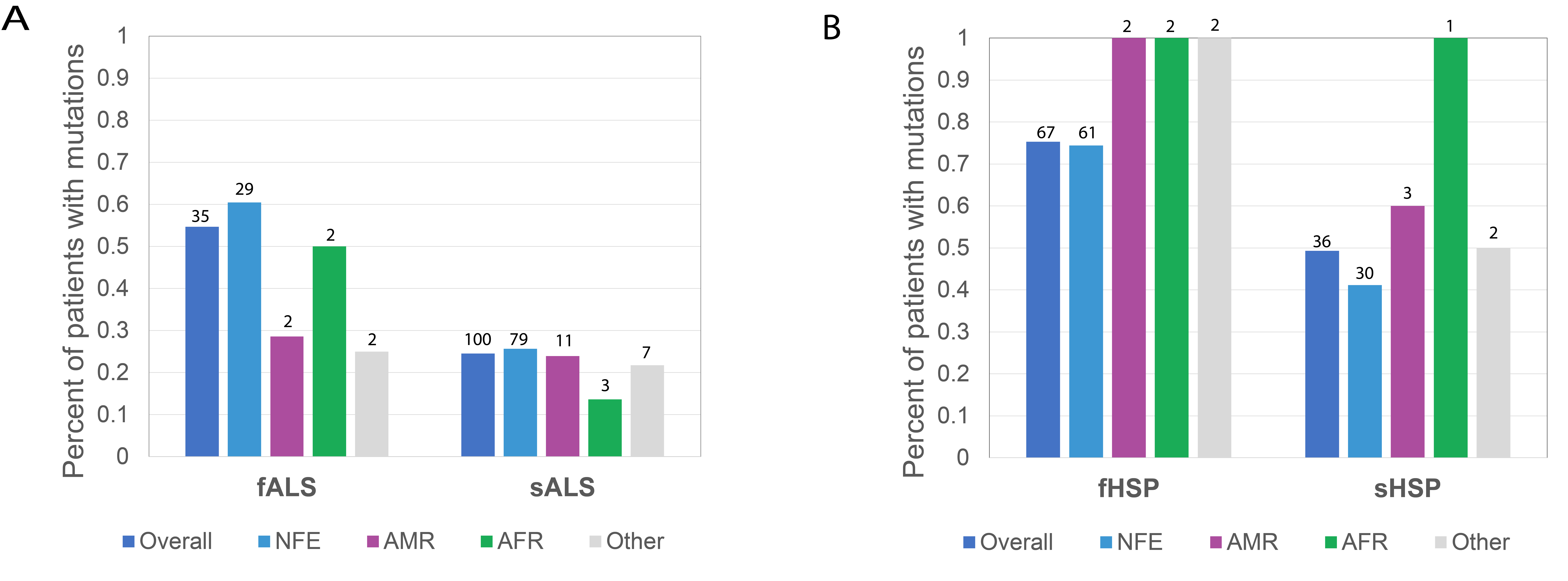


**Figure S7. Pathogenic and predicted disease-mediating URVs in disease-associated genes in ALS and HSP, by ancestry background. (A)** The percentage of familial ALS and sporadic ALS patients carrying any URV. **(B)** The percentage of familial HSP and sporadic HSP patients carrying any URV. The number at the top of each bar indicates the number of patients in the group. AFR – African; AMR – American Indian (Latino); NFE – Non-Finish European.

**
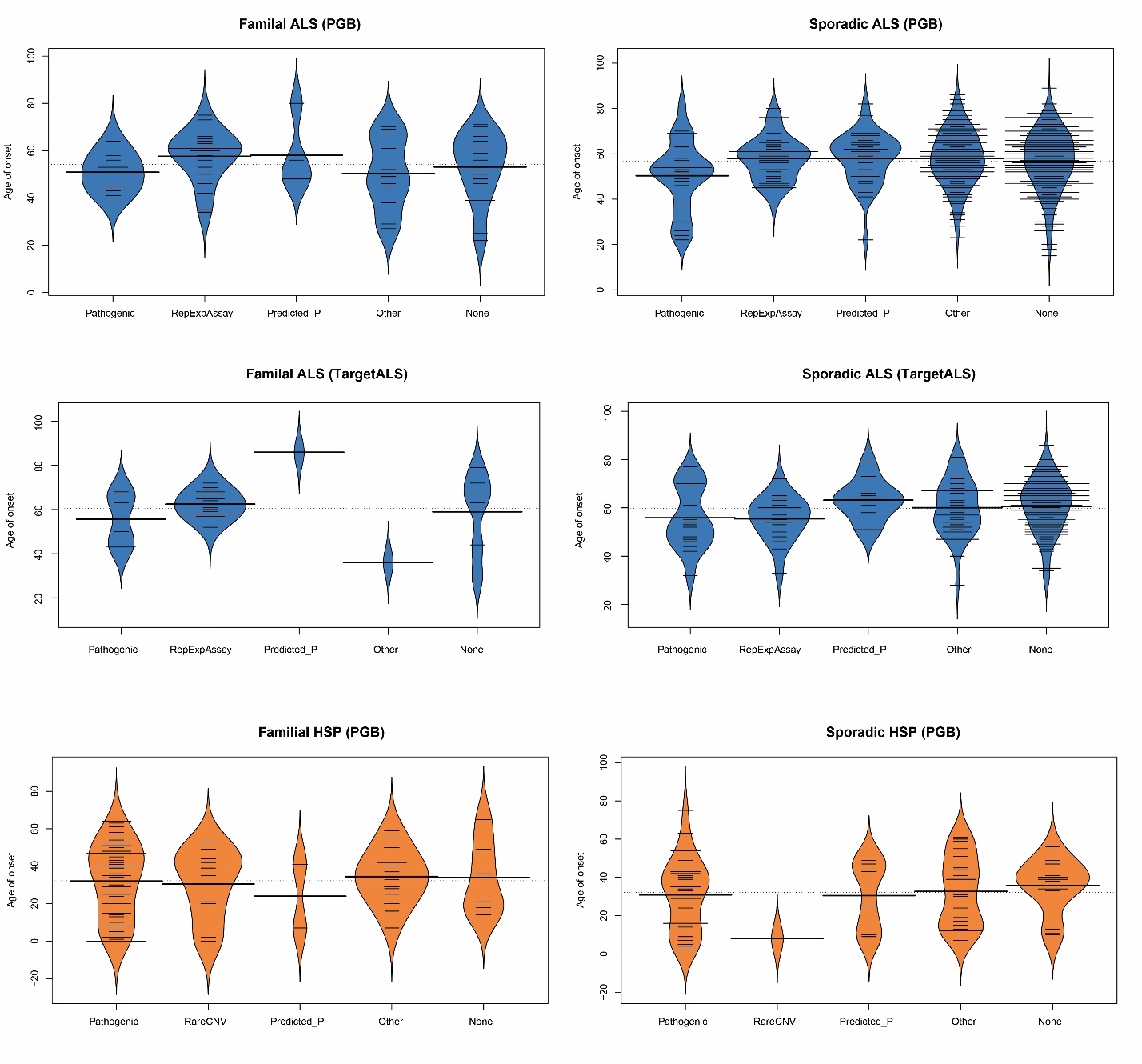
**

**Figure S8 Association of URVs of various categories to age of onset of ALS and HSP.**

**
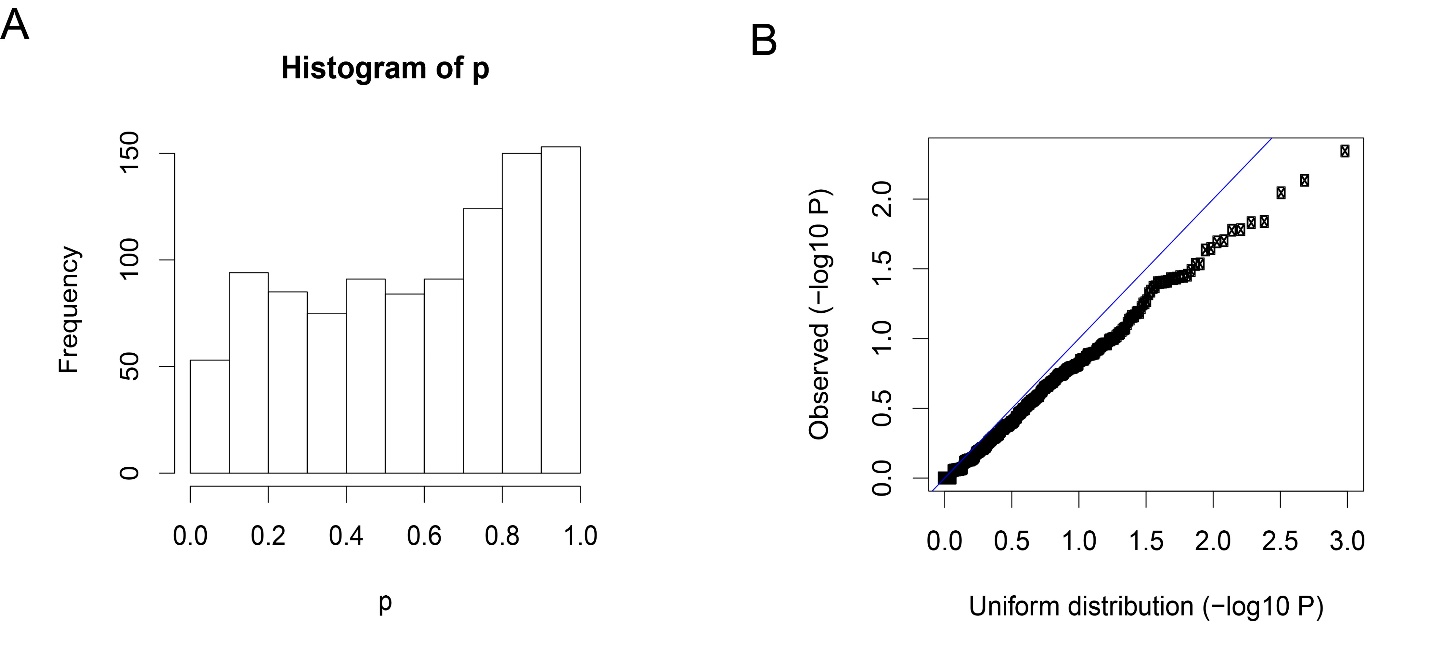
**

**Figure S9. Rare variant burden test to estimate the genetic risk conferred by URVs in randomized gene sets. (A, B)** The distribution and QQ plot of empirical p-values from permutation of 1000 randomly selected list of 60 genes from chr21 to repeat the process against gnomAD. No inflation was observed in the Q-Q plot (B).

**References**

1. Humphrey J, Venkatesh S, Hasan R, Herb JT, de Paiva Lopes K, Kucukali F, et al. Integrative transcriptomic analysis of the amyotrophic lateral sclerosis spinal cord implicates glial activation and suggests new risk genes. Nat Neurosci. 2023;26(1):150-62.

2. Rothstein JD, Svendsen CN, Cudkowicz M, Berry J, Maragakis N, Sherman A, et al. Answer ALS: A Clinical and Comprehensive Multi-Omics Signature for ALS Employing Induced Pluripotent Stem Cell Derived Motor Neurons from 1000 Sporadic and Familial ALS Patients Nationwide. Annals of Neurology. 2016;80:S243-S.

3. Cirulli ET, Lasseigne BN, Petrovski S, Sapp PC, Dion PA, Leblond CS, et al. Exome sequencing in amyotrophic lateral sclerosis identifies risk genes and pathways. Science. 2015;347(6229):1436-41.

4. van der Spek RAA, van Rheenen W, Pulit SL, Kenna KP, van den Berg LH, Veldink JH, et al. The project MinE databrowser: bringing large-scale whole-genome sequencing in ALS to researchers and the public. Amyotroph Lat Scl Fr. 2019;20(5-6):432-40.

5. Chen SW, Francioli LC, Goodrich JK, Collins RL, Kanai M, Wang QB, et al. A genomic mutational constraint map using variation in 76,156 human genomes. Nature. 2024;625(7993).

6. Denny JC, Devaney SA, Gebo KA. The "All of Us" Research Program Reply. New Engl J Med. 2019;381(19):1884-5.

7. DeJesus-Hernandez M, Mackenzie IR, Boeve BF, Boxer AL, Baker M, Rutherford NJ, et al. Expanded GGGGCC hexanucleotide repeat in noncoding region of C9ORF72 causes chromosome 9p-linked FTD and ALS. Neuron. 2011;72(2):245-56.

8. Okonechnikov K, Conesa A, García-Alcalde F. Qualimap 2: advanced multi-sample quality control for high-throughput sequencing data. Bioinformatics. 2016;32(2):292-4.

9. McKenna A, Hanna M, Banks E, Sivachenko A, Cibulskis K, Kernytsky A, et al. The Genome Analysis Toolkit: a MapReduce framework for analyzing next-generation DNA sequencing data. Genome Res. 2010;20(9):1297-303.

10. Danecek P, Bonfield JK, Liddle J, Marshall J, Ohan V, Pollard MO, et al. Twelve years of SAMtools and BCFtools. Gigascience. 2021;10(2).

11. Altshuler DM, Durbin RM, Abecasis GR, Bentley DR, Chakravarti A, Clark AG, et al. A global reference for human genetic variation. Nature. 2015;526(7571):68-+.

12. Conomos MP, Miller MB, Thornton TA. Robust inference of population structure for ancestry prediction and correction of stratification in the presence of relatedness. Genet Epidemiol. 2015;39(4):276-93.

13. Gogarten SM, Sofer T, Chen H, Yu C, Brody JA, Thornton TA, et al. Genetic association testing using the GENESIS R/Bioconductor package. Bioinformatics. 2019;35(24):5346-8.

14. Alexander DH, Novembre J, Lange K. Fast model-based estimation of ancestry in unrelated individuals. Genome Res. 2009;19(9):1655-64.

15. Kopelman NM, Mayzel J, Jakobsson M, Rosenberg NA, Mayrose I. Clumpak: a program for identifying clustering modes and packaging population structure inferences across. Mol Ecol Resour. 2015;15(5):1179-91.

16. Abyzov A, Urban AE, Snyder M, Gerstein M. CNVnator: an approach to discover, genotype, and characterize typical and atypical CNVs from family and population genome sequencing. Genome Res. 2011;21(6):974-84.

17. Chen X, Gupta P, Wang J, Nakitandwe J, Roberts K, Dalton JD, et al. CONSERTING: integrating copy-number analysis with structural-variation detection. Nat Methods. 2015;12(6):527-30.

18. McLeod C, Gout AM, Zhou X, Thrasher A, Rahbarinia D, Brady SW, et al. St. Jude Cloud: A Pediatric Cancer Genomic Data-Sharing Ecosystem. Cancer Discov. 2021;11(5):1082-99.

19. Chen W, Wang S, Tithi SS, Ellison DW, Schaid DJ, Wu G. A rare variant analysis framework using public genotype summary counts to prioritize disease-predisposition genes. Nat Commun. 2022;13(1):2592.

20. Frankish A, Carbonell-Sala S, Diekhans M, Jungreis I, Loveland JE, Mudge JM, et al. GENCODE: reference annotation for the human and mouse genomes in 2023. Nucleic Acids Res. 2023;51(D1):D942-D9.

21. Renton AE, Chio A, Traynor BJ. State of play in amyotrophic lateral sclerosis genetics. Nat Neurosci. 2014;17(1):17-23.

22. Neuenschwander AG, Thai KK, Figueroa KP, Pulst SM. Amyotrophic lateral sclerosis risk for spinocerebellar ataxia type 2 ATXN2 CAG repeat alleles: a meta-analysis. JAMA Neurol. 2014;71(12):1529-34.

23. Demaegd KC, Kernan A, Cooper-Knock J, van Vugt J, Harvey C, Moll T, et al. An observational study of pleiotropy and penetrance of amyotrophic lateral sclerosis associated with CAG-repeat expansion of ATXN2. Eur J Hum Genet. 2025;33(9):1106-12.

24. Douglas AGL. Penetrance and pleiotropy in ATXN2-related amyotrophic lateral sclerosis. Eur J Hum Genet. 2025;33(9):1093-5.

25. Deluca GC, Ebers GC, Esiri MM. The extent of axonal loss in the long tracts in hereditary spastic paraplegia. Neuropathol Appl Neurobiol. 2004;30(6):576-84.

26. Millecamps S, Julien JP. Axonal transport deficits and neurodegenerative diseases. Nat Rev Neurosci. 2013;14(3):161-76.

27. Fischer LR, Culver DG, Tennant P, Davis AA, Wang M, Castellano-Sanchez A, et al. Amyotrophic lateral sclerosis is a distal axonopathy: evidence in mice and man. Exp Neurol. 2004;185(2):232-40.

28. Osmanovic A, Widjaja M, Forster A, Weder J, Wattjes MP, Lange I, et al. SPG7 mutations in amyotrophic lateral sclerosis: a genetic link to hereditary spastic paraplegia. J Neurol. 2020;267(9):2732-43.

29. Daoud H, Zhou S, Noreau A, Sabbagh M, Belzil V, Dionne-Laporte A, et al. Exome sequencing reveals SPG11 mutations causing juvenile ALS. Neurobiol Aging. 2012;33(4):839 e5-9.

30. Filosto M, Piccinelli SC, Palmieri I, Necchini N, Valente M, Zanella I, et al. A Novel Mutation in the Stalk Domain of KIF5A Causes a Slowly Progressive Atypical Motor Syndrome. J Clin Med. 2018;8(1).

31. Simone M, Trabacca A, Panzeri E, Losito L, Citterio A, Bassi MT. KIF5A and ALS2 Variants in a Family With Hereditary Spastic Paraplegia and Amyotrophic Lateral Sclerosis. Front Neurol. 2018;9:1078.

32. Amador MD, Muratet F, Teyssou E, Banneau G, Danel-Brunaud V, Allart E, et al. Spastic paraplegia due to recessive or dominant mutations in ERLIN2 can convert to ALS. Neurol Genet. 2019;5(6):e374.

33. Teyssou E, Chartier L, Amador MD, Lam R, Lautrette G, Nicol M, et al. Novel UBQLN2 mutations linked to amyotrophic lateral sclerosis and atypical hereditary spastic paraplegia phenotype through defective HSP70-mediated proteolysis. Neurobiol Aging. 2017;58:239 e11- e20.

34. Mitsumoto H, Nagy PL, Gennings C, Murphy J, Andrews H, Goetz R, et al. Phenotypic and molecular analyses of primary lateral sclerosis. Neurol Genet. 2015;1(1):e3.

35. Al-Chalabi A, Calvo A, Chio A, Colville S, Ellis CM, Hardiman O, et al. Analysis of amyotrophic lateral sclerosis as a multistep process: a population-based modelling study. Lancet Neurol. 2014;13(11):1108-13.

36. Chio A, Mazzini L, D'Alfonso S, Corrado L, Canosa A, Moglia C, et al. The multistep hypothesis of ALS revisited: The role of genetic mutations. Neurology. 2018;91(7):e635-e42.

37. Ramos EM, Keagle P, Gillis T, Lowe P, Mysore JS, Leclerc AL, et al. Prevalence of Huntington's disease gene CAG repeat alleles in sporadic amyotrophic lateral sclerosis patients. Amyotroph Lateral Scler. 2012;13(3):265-9.

38. Schule R, Wiethoff S, Martus P, Karle KN, Otto S, Klebe S, et al. Hereditary spastic paraplegia: Clinicogenetic lessons from 608 patients. Ann Neurol. 2016;79(4):646-58.

39. Burguez D, Polese-Bonatto M, Scudeiro LAJ, Bjorkhem I, Schols L, Jardim LB, et al. Clinical and molecular characterization of hereditary spastic paraplegias: A next-generation sequencing panel approach. J Neurol Sci. 2017;383:18-25.

40. Depienne C, Fedirko E, Forlani S, Cazeneuve C, Ribai P, Feki I, et al. Exon deletions of SPG4 are a frequent cause of hereditary spastic paraplegia. J Med Genet. 2007;44(4):281-4.

41. Elert-Dobkowska E, Stepniak I, Krysa W, Rajkiewicz M, Rakowicz M, Sobanska A, et al. Molecular spectrum of the SPAST, ATL1 and REEP1 gene mutations associated with the most common hereditary spastic paraplegias in a group of Polish patients. J Neurol Sci. 2015;359(1-2):35-9.

42. Kadnikova VA, Rudenskaya GE, Stepanova AA, Sermyagina IG, Ryzhkova OP. Mutational Spectrum of Spast (Spg4) and Atl1 (Spg3a) Genes In Russian Patients With Hereditary Spastic Paraplegia. Sci Rep. 2019;9(1):14412.

43. Blauw HM, Veldink JH, van Es MA, van Vught PW, Saris CG, van der Zwaag B, et al. Copy-number variation in sporadic amyotrophic lateral sclerosis: a genome-wide screen. Lancet Neurol. 2008;7(4):319-26.

44. Cronin S, Blauw HM, Veldink JH, van Es MA, Ophoff RA, Bradley DG, et al. Analysis of genome-wide copy number variation in Irish and Dutch ALS populations. Hum Mol Genet. 2008;17(21):3392-8.

45. Wain LV, Pedroso I, Landers JE, Breen G, Shaw CE, Leigh PN, et al. The role of copy number variation in susceptibility to amyotrophic lateral sclerosis: genome-wide association study and comparison with published loci. PLoS One. 2009;4(12):e8175.
